# Mediation of pain in the association of sleep problems with falls among older adults in India

**DOI:** 10.1038/s41598-022-27010-3

**Published:** 2023-01-05

**Authors:** T. Muhammad, Priya Maurya, Y. Selvamani, Uma Kelekar

**Affiliations:** 1 International Institute for Population Sciences, Mumbai, Maharashtra 400088 India; 2 SRM Institute of Science and Technology (SRMIST), Chennai, 603203 India; 3 School of Business, College of Business, Innovation, Leadership and Technology, Marymount University, Arlington, VA USA; 4 Marymount Center for Optimal Aging, Marymount University, Arlington, VA USA

**Keywords:** Health care, Geriatrics, Health policy, Public health

## Abstract

Body pain, sleep problems and falls are commonly reported among the elderly population. This study aimed to explore the mediating role of pain in the association of sleep problems with fall-outcomes (falls, fall-injury, and multiple falls) among older adults. Cross-sectional data from the baseline survey of Longitudinal Aging Study in India (LASI), 2017–18 were used. The total sample size for the study was 28,285 older adults aged 60 years and above. Falls and fall-related injuries among older adults in the last two years were self-reported. The Jenkins Sleep Scale (JSS-4) was used to assess sleep problems while pain was assessed using questions on whether respondents reported that they were troubled by pain and they required some form of medication or treatment for the relief of pain. Multivariable logistic regression and mediation analyses were conducted to fulfill the study objectives. While 13% older adults suffered from sleep problems, 38.83% were troubled with pain. Additionally, 12.63%, 5.64% and 5.76% older adults reported falls, fall-injury and multiple falls respectively. Older adults who suffered from sleep problems had higher odds of falls [adjusted odds ratio (aOR): 1.43, confidence interval (CI): 1.30–1.58], fall-injuries, [aOR:1.50,CI:1.30–1.73] and multiple falls [aOR:1.41,CI:1.24–1.62]. Similarly, older adults who were troubled with pain were more likely to report falls [aOR:1.80, CI:1.67–1.95], fall-injuries [aOR:1.66, CI:1.48–1.87] and multiple falls [aOR:1.90,CI:1.69–2.12]. The percent of the mediated effect of pain when examining the association between sleep problems and fall outcomes were reported to be 17.10%, 13.56% and 18.78% in case of falls, fall-injuries and multiple falls respectively. The current study finds evidence that pain mediates the association of sleep problems and falls, fall-injuries, and multiple falls among older Indian adults. Both sleep problems and pain are modifiable risk factors that need attention for fall prevention strategies.

## Introduction

Age-related falls and resulting injuries are a major public health issue which can play a significant role in affecting older adults’ morbidity and mortality^1,2^. Falls are among the leading causes of unintentional mortality worldwide^3^. Globally, based on the World Health Organization (WHO) report, a large proportion of older adults experience falls, with a disproportionate burden of falls-related injuries or mortality reported in low and middle-income countries^4^.

At an individual level, age is a key risk factor for falls^5^. Women experience higher falls than men^6^. Studies also have showed a lower socioeconomic status to be a major risk factor for falls^7^. Health-related factors such as functional limitations, weak handgrip strength, chronic diseases such as diabetes or bone and joint diseases are also associated with a higher prevalence of falls^8–10^. Similarly, sleep problems are a major risk factor for falls. A study conducted among older adults in China found a significant positive association of sleep disturbance and short sleep duration with falls^11^. Another study conducted in six low and middle-income countries showed factors such as depression, sleep problems, multiple chronic conditions and poor cognition to be positively associated with falls-related injury^1^. Another study found similar findings wherein people with sleep problems and pain were more likely to experience falls^12^, while a Finnish study among older adults found the number of chronic conditions to be closely associated with falls^13^.

Falls and fall-related injuries pose major public health threats to an aging society^3,14^ and are scrutinized as one of the 'Geriatric Giants'^15^. Recurrent falls are the leading cause of hospitalization, disability, and mortality^11,16^. The Indian population is aging rapidly with rising longevity^17^. One of the major challenges is to promote well-being and healthy aging of older adults^15,18^. Therefore, there is a growing need for advancing the evidence on the risk factors that contribute to increased morbidity and mortality along with the protective factors that can favor healthy aging, especially in resource-limited settings.

While pain and sleep problems are identified as risk factors for falls, the extent of their contribution to falls is unclear in the literature. To the best of our knowledge, no study has examined the mediating effect of pain on the relationship between sleep and falls or fall-related injuries or multiple falls among older adults in India. While falls and fall-related consequences are higher in low and middle-income countries, it is important to understand the role of sleep and pain and their effect on falls and fall-related injuries. In old age, pain and sleep problems are common risk factors leading to adverse health outcomes in India. A growing body of literature suggest that pain is associated with poor self-rated health^19^, depression^20^, and lower quality of life^19^. Through this study, any additional evidence of the role of pain in the association of sleep problems with fall outcomes that we find will be helpful in guiding policy-makers in developing interventions and policies to prevent falls and falls-related injuries among its elderly population.

Thus, the objectives of the current study are (1) to examine the association of pain and sleep problems with falls and falls-related injury (2) to study the direct and indirect effect of sleep problems on falls, falls-related injuries and multiple falls, and (3) to see the mediating role of pain, using a nationally representative dataset that comprises community-dwelling older adults in India.

## Methods

### Data

#### Study participants

**Figure 1:**
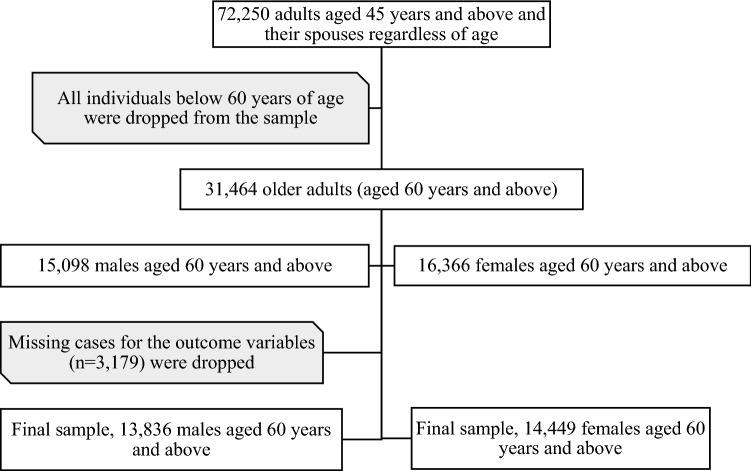
Sample selection criteria for this study.

The current study is based on the data from the baseline survey of the Longitudinal Aging Study in India (LASI), 2017–18. The LASI is a national-representative survey of 72,250 individuals aged 45 years and above and their spouses regardless of age across all states and union territories of the country. The survey provides important information on the physical, psychological, cognitive and social health of the older adult population in India. In the LASI, the sample selection is based on a multistage stratified cluster sample design, including a three-stage sampling design in rural areas and a four-stage sampling design in urban areas. The survey was administered face-to-face by an interviewer during household visits using computer-assisted personal interview (CAPI) technology in the local language. The total response rate at individual level was 95.6%. The details of sampling design, survey instruments, and data collection procedures are published elsewhere^21^. The Indian Council of Medical Research (ICMR) extended the necessary guidance and ethical approval for conducting the LASI. All the methods were carried out in accordance with those relevant guidelines and regulations. Prior informed consent from the respondents was taken before conducting the interviews. Our study sample is limited to eligible respondents aged 60 years and above. The missing cases for the outcome variable (n = 3179) were dropped from the analysis, resulting in a final sample size of 28,285 (13,836 males and 14,449 females) older adults aged 60 years and above. The sample selection procedure for this study is summarized in Fig. 1.

### Measures

#### Outcome variables

Falls among older adults in the last two years were self-reported and assessed using the question, *'In the past two years, have you fallen down?*' The responses were coded as ‘no’ and ‘yes’. Further, fall-related injuries were assessed by the following survey question that ‘*In that fall, did you injure yourself seriously enough to need medical treatment?’* and the responses were similarly codes as ‘no’ and ‘yes’. Additionally, information on multiple falls was calculated from the question on number of falls in the last two years. Those who reported falls > 1 were classified as having multiple falls.

#### Main exposure variable

Sleep problems were assessed using the following four questions in the LASI survey, adapted from the Jenkins Sleep Scale (JSS-4)^22^: “*How often do you have trouble falling asleep*?” (1) “*How often do you have trouble with waking up during the night?*” (2) “*How often do you have trouble with waking up too early and not being able to fall asleep again?*” (3) “*How often did you feel unrested during the day regardless of the number of hours of sleep you had?*” These questions address the respondents’ sleep during the past one month. Response options were “never, rarely (1–2 nights per week), occasionally (3–4 nights per week), and frequently (5 or more nights per week)” (item four was reverse coded). The presence of sleep problems was “coded as 1 if people responded ‘frequently’ to any of the four aforementioned questions^23^. The JSS-4 scale of assessing sleep problems has been used in clinical settings^24^, epidemiological cohort studies^25,26^ and observational studies on normal healthy people^27^. The scale has proven to have excellent reliability and demonstrated good construct validity^28^. Additionally, it has been validated in the Indian setting with local languages^29^. The internal consistency of JSS-4 (Chronbach’s alpha) was 0.87 in our study.

#### Mediator variable

In this study, pain was the mediating variable in the association between sleep problems and falls, fall-injury and multiple falls. Pain was self-reported by older individuals, and assessed if respondents reported that they were troubled by pain and required some form of medication or treatment for the relief of pain, with the help of the following question ‘*Are you often troubled with pain?*, and coded as no and yes^20^.

#### Other covariates

Socio-demographic variables included age (recoded as 60–69, 70–79 and 80 +), sex (male and female), education (no education, primary education and secondary/higher education), marital status (married, widowed and others which included separated, divorced and never married), living arrangements (living alone, with spouse, with spouse and children and others) and work status (never worked, currently working, not working and retired). Self-rated health was recoded as good (Very good, good and fair) and poor (poor and very poor). The activities of daily living (ADL) is a term used to refer to normal daily self-care activities (such as movement in bed, changing position from sitting to standing, feeding, bathing, dressing, grooming, personal hygiene etc.) The ability or inability to perform ADLs is used to measure a person’s functional status, especially in the case of people with disabilities and older adults^30,31^. Instrumental activities of daily living (IADL) that are not necessarily related to the fundamental functioning of a person, but letting an individual live independently in a community, are used as functional health indicator. In this study, difficulty in ADL and IADL were coded as binary variables (no and yes) representing the presence of at least one difficulty.

Further, the monthly per capita consumption expenditure (MPCE) quintile was assessed using household consumption data. Food expenditure was collected based on a reference period of seven days, and non-food expenditure was collected based on reference periods of 30 days and 365 days. Food and non-food expenditures have been standardized to the 30-day reference period. The MPCE is computed and used as the summary measure of consumption. The variable was then divided into five quintiles i.e., from poorest to richest. Religion was coded as Hindu, Muslim, Christian, and Others. Caste was recoded as Scheduled Caste/Scheduled Tribe (SC/ST), Other Backward Classes (OBC), and others. The SC refers to the population that is socially segregated and financially/economically weak by their lower status as per the caste hierarchy. Similarly, the ST refers to the indigenous populations who are amongst the most disadvantaged and discriminated socio-economic groups in the country. The OBC is the group of people identified as ‘socioeconomically and educationally backward’ while the ‘other’ caste category is identified as having higher social status, mostly those belonging to an upper caste group^32^. Place of residence was coded as urban and rural. Also, region of the country was coded as North, Central, East, Northeast, West, and South.

### Statistical analysis

Descriptive statistics along with results of cross-tabulations are presented in the study. Additionally, multivariable logistic regression analysis^33^ was conducted to find out the association between the outcome variables (falls, fall-injury and multiple falls) and sleep problems. The estimates were presented in the form of odds ratio (OR) along with a 95% confidence interval (CI). Additionally, the total effect in the observed association was divided into direct (the association of sleep problems with fall, fall-injury and multiple falls after controlling for all the covariates) and indirect or mediating effects (the association of sleep problems with fall variables through pain) using Karlson–Holm–Breen (KHB) method^34,35^. The KHB method is a recently developed method for assessing mediating effects that allow total effects to be divided into direct and indirect (i.e., mediation) effects for both discrete and continuous variables. The mediation percentage (the indirect effect divided by the total effect) is interpreted as the percentage of the association explained by the mediator variable. All statistical models were adjusted for covariates including age, sex, education, marital status, living arrangements, work status, self-rated health, ADL/IADL difficulty, MPCE quintiles, religion, caste, place of residence and region. The statistical analysis was performed using Stata 15.1. Individual weights were used to make the estimates nationally representative.

### Ethical approval

The Indian Council of Medical Research (ICMR) extended the necessary guidance, guidelines and ethical approval for conducting the LASI survey. All methods were carried out in accordance with those relevant guidelines and regulations. The survey agencies that conducted the field survey for the data collection have collected prior consent (signed and oral) for both the interviews and biomarker tests from the eligible respondents in accordance with the Human Subjects Protection.

## Results

### Sample characteristics

Table 1 presents the socioeconomic and health profile of older adults in the current study. A proportion of 13.12% older adults suffered from sleep problems and 38.83% older adults were troubled with pain in the study. More than 10% of the participants were aged 80 years and above and the sample included 51.08% female respondents. A proportion of 33.25% older adults were widowed in the study, while 5.06% older adults were living alone. More than half of the participants had no formal education and 29.08% were currently working. Furthermore, 21.70% older adults had poor self-rated health, 20.11% and 42.96% had ADL and IADL difficulty respectively. Besides, 65.42% of the sample lived in rural areas of the country.

**Table 1:** Sample distribution and rates of fall, fall-injury and multiple falls by background characteristics among older adults.

Variables	Distribution		Fall		Fall-injury		Multiple falls	
	Frequency	w col %	w%	*p*-value	w%	*p*-value	w%	*p*-value
**Sleep problems**				< 0.001		< 0.001		< 0.001
No	24,567	86.88	11.58		5.11		5.22	
Yes	3709	13.12	19.12		8.95		9.04	
**Pain**				< 0.001		< 0.001		< 0.001
No	17,293	61.17	9.32		4.09		3.99	
Yes	10,977	38.83	18.08		8.19		8.66	
**Age (in years)**				0.047		0.679		< 0.001
60–69	17,179	60.74	12.42		5.76		5.33	
70–79	8172	28.89	12.52		5.03		6.08	
80 +	2934	10.37	14.12		6.68		7.21	
**Sex**				< 0.001		< 0.001		< 0.001
Male	13,836	48.92	11.69		5.28		4.91	
Female	14,449	51.08	13.52		5.98		6.56	
**Marital status**				< 0.001		0.001		< 0.001
Currently in union	18,110	64.03	12.04		5.37		5.21	
Widowed	9404	33.25	13.87		6.22		6.87	
Others	771	2.73	9.96		4.33		3.58	
**Living arrangement**				< 0.001		0.001		< 0.001
Alone	1431	5.06	14.78		5.76		8.31	
With spouse	5627	19.89	12.62		5.82		5.34	
With spouse and children	12,259	43.34	11.62		5.06		5.08	
Others	8968	31.71	13.56		6.26		6.44	
**Educational status**				< 0.001		< 0.001		< 0.001
None	15,036	53.16	13.16		5.91		6.47	
Primary	5218	18.45	13.9		6.40		5.80	
Secondary/higher	8031	28.39	10.63		4.55		4.18	
**Work status**				< 0.001		0.001		< 0.001
Never worked	7777	27.50	11.3		4.84		5.31	
Not working	9769	34.54	13.43		5.80		6.56	
Working	8225	29.08	13.41		6.31		5.56	
Retired	2514	8.89	10.11		4.88		4.23	
**Self-rated health**				< 0.001		< 0.001		< 0.001
Good	21,753	78.30	11.36		5.16		4.76	
Poor	6030	21.70	16.86		7.29		8.78 lePara>	
**ADL difficulty**				< 0.001		< 0.001		< 0.001
No	22,570	79.89	11.78		5.22		5.18	
Yes	5682	20.11	15.6		7.12		7.73	
**IADL difficulty**				< 0.001		< 0.001		< 0.001
No	16,095	57.04	11.16		4.95		4.78	
Yes	12,121	42.96	14.3		6.43		6.83	
**MPCE quintile**				0.031		0.031		0.741
Poorest	5876	20.77	11.5		4.44		5.38	
Poorer	5834	20.63	13.64		5.88		6.17	
Middle	5746	20.31	11.86		5.53		5.59	
Richer	5524	19.53	12.72		5.52		6.00	
Richest	5305	18.76	13.67		7.24		5.62	
**Caste**				0.001		0.160		< 0.001
SC/ST	9371	33.13	13.26		6.34		5.61	
OBC	10,621	37.55	11.71		4.89		5.83	
Others	8,293	29.32	13.5		6.18		5.78	
**Religion**				< 0.001		< 0.001		0.217
Hindu	20,567	72.71	12.59		5.66		5.70	
Muslim	3350	11.84	12.28		5.65		5.42	
Others	4368	15.44	13.67		5.41		7.01	
**Place of residence**				< 0.001		< 0.001		< 0.001
Urban	9781	34.58	10.35		4.62		4.77	
Rural	18,504	65.42	13.57		6.06		6.16	
**Region**				< 0.001		< 0.001		< 0.001
North	5318	18.80	10.25		4.65		4.62	
Central	3829	13.54	13.79		6.01		7.09	
East	4860	17.18	15.47		7.12		6.50	
Northeast	3469	12.26	9.89		4.57		4.15	
South	6905	24.41	8.44		2.80		4.43	
West	3904	13.80	15.16		7.83		5.98	
Total	28,285	100	12.63		5.64		5.76	

Notes: w col %: Weighted column percentage; w%: weighted percentage prevalence to account for survey design and provide national population estimates; ADL: Activities of daily living; IADL: Instrumental activities of daily living; MPCE: Monthly per capita consumption expenditure.

Prevalence of falls, fall-related injury and multiple falls is estimated to be 12.63%, 5.64% and 5.76% respectively. Older adults who suffered from sleep problems had a higher prevalence (19.12% vs. 11.58%) of falls than those did not suffer from sleep problems. Similarly, higher prevalence of a fall-related injury (8.95% vs. 5.11%) and multiple falls (9.04% vs. 5.22%) were reported among those who had sleep problems. Besides, 18.08% of older adults who were troubled with pain reported falls in comparison to those who had no pain (9.32%). Consistently, fall-related injury (8.19% vs. 4.09) and multiple falls (8.66% vs. 3.99%) were observed to be higher among those who reported pain.

Table 2 presents the state-wise prevalence of falls, fall-related injury and multiple falls. The higher prevalence was observed in the states of Odisha (23.13%), Punjab (20.83%) and Kerala (18.70%). The higher prevalence of fall-related injuries was observed in the states of Punjab (9.94%), Gujarat (8.70%) and Odisha (7.80%). Besides, higher prevalence of multiple falls was found in the states of Kerala (12.24%), Punjab (11.44%) and Odisha (10.62%).

**Table 2:** State-wise prevalence (%) of falls, fall-injury and multiple falls among older adults (60 + years), India, LASI Wave 1, 2017–18.

State/UT	Fall	Fall-injury	Multiple falls
	%	%	%
Jammu & Kashmir	3.41	1.17	0.29
Himachal Pradesh	12.13	3.69	6.70
Punjab	20.83	9.94	11.44
Chandigarh	9.93	5.36	1.83
Uttarakhand	12.51	3.74	5.79
Haryana	12.79	5.76	5.03
Delhi	8.71	5.02	5.03
Rajasthan	6.23	3.05	2.22
Uttar Pradesh	16.03	6.92	8.46
Bihar	14.77	7.54	7.44
Arunachal Pradesh	10.11	0.45	5.17
Nagaland	4.15	0.10	3.03
Manipur	4.02	2.22	0.86
Mizoram	1.61	0.95	0.07
Tripura	11.22	4.08	5.47
Meghalaya	3.20	0.92	1.89
Assam	12.24	6.25	4.96
West Bengal	11.97	6.24	3.75
Jharkhand	16.87	7.37	5.44
Odisha	23.13	7.80	10.62
Chhattisgarh	11.85	4.20	6.03
Madhya Pradesh	10.36	4.87	4.97
Gujarat	17.04	8.70	7.07
Daman & Diu	13.25	7.15	5.02
Dadra & Nagar Haveli	17.49	8.12	6.88
Maharashtra	14.37	7.49	5.51
Andhra Pradesh	6.03	3.15	3.29
Karnataka	7.02	2.65	3.32
Goa	10.79	3.34	6.07
Lakshadweep	2.65	1.42	2.20
Kerala	18.70	3.33	12.24
Tamil Nadu	9.35	2.61	4.88
Puducherry	6.33	2.64	3.51
Andaman & Nicobar Islands	4.67	2.56	2.78
Telangana	5.08	2.74	1.48
India	12.63	5.64	5.76

Table 3 presents the results of multivariable logistic regression. Older adults who suffered from sleep problems had significantly higher odds of falls [aOR: 1.43, CI: 1.30–1.58], fall-related injury [aOR: 1.50, CI: 1.30–1.73] and multiple falls [aOR: 1.41, CI: 1.24–1.62]. Similarly, older adults who were troubled with pain were more likely to report falls [aOR: 1.80, CI: 1.67–1.95], fall-related injury [aOR: 1.66, CI: 1.48–1.87] and multiple falls [aOR: 1.90, CI: 1.69–2.12].

**Table 3:** Multivariable logistic regression estimates of fall, fall-injury and multiple falls by socioeconomic and health characteristics among older adults.

Variables	Fall	Fall-injury	Multiple falls
	aOR (95% CI)	aOR (95% CI)	aOR (95% CI)
**Sleep problems**			
No	Ref.	Ref.	Ref.
Yes	1.43*** (1.30–1.58)	1.50*** (1.30–1.73)	1.41*** (1.24–1.62)
**Pain**			
No	Ref.	Ref.	Ref.
Yes	1.80*** (1.67–1.95)	1.66*** (1.48–1.87)	1.90*** (1.69–2.12)
**Age (in years)**	1.00 (0.99–1.00)	0.99 (0.99–1.00)	1.00 (1.00–1.01)
**Sex**			
Male	Ref.	Ref.	Ref.
Female	1.31*** (1.18–1.44)	1.26** (1.09–1.46)	1.45*** (1.26–1.67)
**Marital status**			
Currently in union	Ref.	Ref.	Ref.
Widowed	0.85 (0.57–1.26)	0.69 (0.41–1.15)	0.80 (0.47–1.37)
Others	0.71 (0.45–1.13)	0.62 (0.33–1.16)	0.51* (0.26–1.00)
**Living arrangement**			
Alone	Ref.	Ref.	Ref.
With spouse	0.79 (0.52–1.20)	0.62 (0.35–1.08)	0.68 (0.38–1.21)
With spouse and children	0.83 (0.55–1.26)	0.68 (0.39–1.17)	0.75 (0.43–1.33)
Others	1.08 (0.91–1.28)	1.15 (0.89–1.49)	0.94 (0.75–1.18)
**Education (in years)**	1.00 (0.99–1.01)	1.00 (0.98–1.01)	0.99 (0.98–1.01)
**Work status**			
Never worked	Ref.	Ref.	Ref.
Not working	1.30*** (1.17–1.45)	1.28** (1.09–1.50)	1.35*** (1.17–1.56)
Working	1.43*** (1.27–1.61)	1.46*** (1.22–1.74)	1.46*** (1.23–1.73)
Retired	0.98 (0.81–1.18)	1.09 (0.83–1.42)	1.04 (0.79–1.37)
**Self-rated health**			
Good	Ref.	Ref.	Ref.
Poor	1.26*** (1.15–1.38)	1.11 (0.97–1.27)	1.49*** (1.32–1.68)
**ADL difficulty**			
No	Ref.	Ref.	Ref.
Yes	1.18** (1.07–1.30)	1.18* (1.03–1.37)	1.27*** (1.11–1.45)
**IADL difficulty**			
No	Ref.	Ref.	Ref.
Yes	1.16*** (1.07–1.27)	1.21** (1.06–1.37)	1.14* (1.00–1.28)
**MPCE quintile**			
Poorest	Ref.	Ref.	Ref.
Poorer	1.20** (1.07–1.35)	1.25* (1.04–1.50)	1.10 (0.93–1.30)
Middle	1.10 (0.97–1.24)	1.27* (1.05–1.52)	1.10 (0.93–1.31)
Richer	1.30*** (1.15–1.47)	1.53*** (1.27–1.84)	1.25* (1.05–1.49)
Richest	1.29*** (1.13–1.46)	1.67*** (1.37–2.02)	1.22* (1.02–1.46)
**Religion**			
Hindu	Ref.	Ref.	Ref.
Muslim	0.84** (0.74–0.96)	0.92 (0.76–1.10)	0.92 (0.77–1.10)
Others	1.18** (1.04–1.34)	0.91 (0.75–1.11)	1.47*** (1.24–1.74)
**Caste**			
SC/ST	Ref.	Ref.	Ref.
OBC	1.19*** (1.07–1.31)	1.05 (0.91–1.22)	1.31*** (1.14–1.51)
Others	1.16** (1.04–1.30)	1.06 (0.90–1.24)	1.22* (1.04–1.42)
**Place of residence**			
Urban	Ref.	Ref.	Ref.
Rural	1.22*** (1.11–1.33)	1.13 (0.99–1.30)	1.28*** (1.12–1.46)
**Region**			
North	Ref.	Ref.	Ref.
Central	1.24** (1.08–1.42)	1.26* (1.03–1.55)	1.39*** (1.15–1.69)
East	1.55*** (1.37–1.75)	1.64*** (1.37–1.97)	1.42*** (1.18–1.70)
Northeast	0.64*** (0.54–0.75)	0.61*** (0.46–0.79)	0.66*** (0.52–0.85)
West	0.82** (0.72–0.93)	0.69*** (0.56–0.84)	1.01 (0.84–1.21)
South	1.37*** (1.20–1.57)	1.55*** (1.29–1.88)	1.28* (1.05–1.55)

Notes: *if *p*-value < 0.05, ** if *p*-value < 0.005, *** if *p*-value < 0.001; aOR: adjusted Odds Ratio; ADL: Activities of daily living; IADL: Instrumental activities of daily living; MPCE: Monthly per capita consumption expenditure.

Table 4 presents the results from the mediation analysis. Older adults who suffered from sleep problems had higher odds of falls [aOR: 1.54, CI: 1.40–1.70], fall-related injury [aOR: 1.60, CI: 1.39–1.84] and multiple falls [aOR: 1.53, CI: 1.34–1.75]. The associations of sleep problems with fall outcomes were mediated by pain. The percent of mediated effects were reported to be 17.10%, 13.56% and 18.78% in case of falls, fall-related injuries and multiple falls respectively.

**Table 4:** Direct and indirect effects of sleep problems on falls, fall-related injuries, and multiple falls.

	Fall	Fall-injury	Multiple falls
	aOR (95% CI)	aOR (95% CI)	aOR (95% CI)
**Sleep problems**			
Total effect	1.54*** (1.40–1.70)	1.60*** (1.39–1.84)	1.53*** (1.34–1.75)
Direct effect	1.43*** (1.30–1.58)	1.50*** (1.30–1.73)	1.41*** (1.24–1.62)
Indirect effect via pain	1.08*** (1.06–1.09)	1.07*** (1.05–1.08)	1.08*** (1.06–1.10)
PEM (in %)	17.10	13.56	18.78

Notes: aOR: OR adjusted for age, sex, education, marital status, living arrangements, self-rated health, ADL/IADL difficulty, MPCE quintiles, religion, caste, place of residence and regions; PEM: Percent of effect mediated.

## Discussion

Although sleep problems have been identified as a risk factor for falls, whether this association is mediated by pain is unclear in the current literature. Our study found that a large number of older Indian adults are suffering from sleep problems that can potentially have serious consequences on their health and overall wellbeing^36–38^. This study further unfolds the role of pain in the relationship between sleep problems and falls, fall-related injuries, and multiple falls. It demonstrates the mediator effect of pain in the relationship between sleep problems and falls and related injuries. In line with previous studies, the association of sleep problems and pain with falls in the geriatric population was found to be positive and statistically significant^39–42^.

A significant proportion of older people (12.63%) reported falls in the past two years, which appears to be lower than estimates reported in studies in other countries^43–47^. A systematic review from India reported that a pooled prevalence of falls was 31% among older people^48^. Similarly, Pichai et al. (2019) found one-fourth of their study participants to have a history of falling^46^. However, the prevalence of falls, related-injuries and multiple falls in our study was similar to previous evidence from India^18,47^. One plausible explanation for the dissimilarity in prevalence in other studies could be that reporting of falls and related injuries was associated with hospitalization^46^. Older adults who experienced hospitalization with falls and were institutionalized will be more likely to recall falls and the LASI survey did not collect information from older adults who were institutionalized. Therefore, given that multiple falls and injuries are an indication of poor physical and cognitive health of elderly people^42,44^, preventing falls among its rising older adult population remains a major public health concern in India.

Sleep problems in late life are common among older people and are associated with declining physical activity, functional impairment, and decreased quality of life^14,39,49^. Our study found 13.12 percent of the geriatric population to have reported any sleep problems. This percent is lower than the estimates obtained by other hospital-based studies among older adults that reported an insomnia prevalence to range between 11.8% and 32%^50^. While our study is a population-based study representative of the older adult population in the whole country, the lower prevalence might also have been on account of older people in the community-dwellings cognizing sleep problems as a natural part of aging, thus leading to its under-estimation^43,49,51^. Our study also found that older people with reported sleep problems had a significant increase in the risk of falls, fall-related injuries and multiple falls, which was consistent with other studies^39,52,53^. Older people with reduced psychomotor performance, sleep problems, short sleep duration in night and naps are at higher risk of falls and related injuries^54^. This possible delineation by which sleep problems affect falls occurrence is due to daytime sleepiness, cognitive disorder, impaired standing balance and walking ability^53,55^. In particular, Morelhão et al.^39^ endorsed that sleep deprivation causes detrimental damage in an organism disrupting the systems of balance and coordination, difficulty in sustaining attention and concentration, leading to falls, related injuries, and multiple falls in old age^39^. These results emphasized the importance of sleep problems in the obviation of falls and related injuries among older people. However, the relationship between sleep problems and falls was significantly attenuated when pain was controlled. Thus, our study indicated that the presence of pain might be a pathway in the association between self-reported sleep problems and falls among older people.

Consistent with prior evidence^16,40,41,56^, our findings indicate that older adults with pain experienced more falls, related injuries and multiple falls than those without pain. A meta-analysis study conducted by Stubbs et al. (2014) revealed that older people who reported pain were 56% at higher risk of falls compared to those with no pain. Furthermore, they also demonstrated that half of the study participants with pain reported one or more falls^16^. A 4-year follow-up study among community-dwelling older adults aged 70 years and above revealed that pain interference and pain distribution independently predicted injurious falls^57^. Another 1-year follow-up study found that self-reported back pain was associated with recurrent falls among older persons^58^. The mechanism behind this significant association could be multifaceted, namely, local joint pathology, the neuromuscular effect of pain and central mechanisms, through which pain mediates with cognitive function as age advances^41^. Additionally, factors such as fear of falling, history of falls and fall efficiency are related to falling, increase the risk of falls, and are associated with pain^59,60^. Future studies are warranted to examine these aspects.

Both sleep problems and pain serve as a common array of physical and mental health comorbidities^56,61^. However, no studies have shed light on the mediation effect of pain in the relationship between sleep problems and falls, falls-related injuries and multiple falls among Indian older adults. The studies based on medical interventions demonstrated that the onset of pain appears as a side effect that coexists with the development of sleep disturbances or the other way round^61,62^. Our findings also indicated that 17.10% of falls, 13.56% of falls injuries, and 18.78% of recurrent falls were mediated by pain among older people who had sleep problems. One mechanism behind this association is that sleep problems expand the risk for the genesis of pain among older people, which eventually worsens the long term prognosis of existing pain^61^ and influences mobility and functionality in day to day life resulting in a higher incidence of falls among older adults^14,48^. Studies also propound that sleep problems can lead to systemic inflammation in the body that escalate pain^59^ which further prognosticate poorer physical and psychological functioning eventuating in falls and injuries^59,60^. Another possible explanation could be that older adults with sleep problems will amplify chronic back pain. Also, sleep deficiency disrupts the body's immune response and body's cognitive functions. Sleeping difficulty hampers the pain and pain makes sleeping more difficult^63^. Therefore, the bidirectional relationship can cause problems in concentration and attention, thereby increasing the risk of falling and multiple falls^55^.

The present study also found that falls, falls-related injury and multiple falls were associated with other factors, including socio-demographic and physiological characteristics of individuals. These factors can either relate to falls independently or act in conjunction with other potential risk factors to impact on the prevalence of falls among older adults. Furthermore, the female gender was at higher risk of falls which was also identified in previous studies^15,18^. This may be partially explained by genetic factors, lifestyle demands and differences in bone density between men and women^10,18^. Older people having difficulties in ADL and IADL were at a higher risk of falls and injuries in our study. Consistently, ADL and IADL related difficulties significantly predicted falls in previous studies^15,59^. Thus, enhancing physical activity and improving functional ability can probably reduce the risk of falls among older people^47,64^.

## Strengths and limitations

The major strength of the study is the large nationally-representative survey data with comprehensive information on socioeconomic and health characteristics of older population. The study included a large number of potential confounders that strengthen the current findings. Although the present study provides evidence on the role of pain in sleep problems and associated falls, related injuries, and multiple falls, it has several limitations. The study deals with self-reported information resulting in recall bias and under-reporting. While falls are typically reported using a single-year time-frame or less, the 2-year time-frame used in LASI might be associated with some recall bias on the part of the respondents. However, LASI follows other longitudinal aging studies in using the timeframe for fall outcomes such as the English Longitudinal Study of Ageing (ELSA)^65^. Also, there was no definition in the questionnaire of what constituted a fall. Studies have differed in how a fall is defined and this diversity may not facilitate a direct comparison of findings^66^. The data were cross-sectional in nature, thus limiting the interpretation of causal association. Sleep problems was assessed through many questions, but there is great diversity in sleep patterns depending on cultural, contextual, and structural factors. For instance, some people take quality sleep in less time and others getting regular sleep hours have sleep problems. Therefore, sleep quality and duration are also necessary to explore the relationship between sleep and falls.

## Conclusion

The current study comes up with evidence that pain mediates the association of sleep problems and falls, related injuries, and multiple falls among older Indian adults. Both sleep problems and pain are modifiable risk factors that need attention for fall prevention strategies. From an economic standpoint, falls are costly to treat and due to lack of health insurance, non-affordability is a common cause for not seeking any medical treatment among older adults^67^. Therefore, policy-makers can consider development of falls prevention programs. These programs include education, exercises for balance and strength training, medication review, home safety modifications, correction of refractive errors etc. and several have shown to be effective in improving balance and mobility in the Indian context^68–71^. Encouraging physical exercise and social participation should be prioritized to prevent or reduce the incidence of falls and injuries related to falling among older people. Measures to improve sleep health and reducing pain through nutritional intervention such as vitamin-D supplementation will be useful. Lastly, developing educational programs and providing appropriate facilities such as several forms of care-giving assistance for disabled senior citizens in the neighbourhood might be some cost-effective interventions to also consider implementing.

## Data Availability

The study uses secondary data which is available on reasonable request through https://www.iipsindia.ac.in/content/lasi-wave-i. The data are also available at The Gateway to Global Aging Data https://www.g2aging.org/
